# Heterogeneous Ice Nucleation by Soufriere Hills Volcanic Ash Immersed in Water Droplets

**DOI:** 10.1371/journal.pone.0169720

**Published:** 2017-01-05

**Authors:** T. P. Mangan, J. D. Atkinson, J. W. Neuberg, D. O’Sullivan, T. W. Wilson, T. F. Whale, L. Neve, N. S. Umo, T. L. Malkin, B. J. Murray

**Affiliations:** 1 School of Earth and Environment, University of Leeds, Leeds, United Kingdom; 2 School of Chemistry, University of Leeds, Leeds, United Kingdom; University of Oxford, UNITED KINGDOM

## Abstract

Fine particles of ash emitted during volcanic eruptions may sporadically influence cloud properties on a regional or global scale as well as influencing the dynamics of volcanic clouds and the subsequent dispersion of volcanic aerosol and gases. It has been shown that volcanic ash can trigger ice nucleation, but ash from relatively few volcanoes has been studied for its ice nucleating ability. In this study we quantify the efficiency with which ash from the Soufriere Hills volcano on Montserrat nucleates ice when immersed in supercooled water droplets. Using an ash sample from the 11^th^ February 2010 eruption, we report ice nucleating efficiencies from 246 to 265 K. This wide range of temperatures was achieved using two separate droplet freezing instruments, one employing nanolitre droplets, the other using microlitre droplets. Soufriere Hills volcanic ash was significantly more efficient than all other ash samples that have been previously examined. At present the reasons for these differences are not understood, but may be related to mineralogy, amorphous content and surface chemistry.

## Introduction

Water droplets in tropospheric clouds can supercool to below 236 K before freezing homogeneously, but suitable aerosol particles can catalyse ice formation at much higher temperatures. These ice nucleating particles (INPs) can have wide ranging effects on the properties of clouds, which has an indirect impact on climate [1]. There are many INP types in the Earth’s atmosphere, but it is currently unclear which are most important in different regimes [2,3].

One category of INP is volcanic ash which may have a sporadic impact on clouds as far as thousands of kilometres from the volcano [4] and has been linked to sporadically high concentrations of INP in the vicinity of volcanoes [5]. Within a volcanic ash influenced air mass it is thought that ash might compete with other important INP types, such as desert dusts [2]. In addition to impacting clouds on a regional scale, volcanic ash is the primary component of volcanic clouds and plumes emitted from erupting volcanoes. Durant et al. [6] suggest that volcanic clouds will be INP-rich in contrast to ‘meteorological’ clouds which are often INP-limited and that this will produce clouds with very different microphysics. Supercooled water forming in the volcanic cloud may freeze well above the homogeneous limit due to the presence of the ash, resulting in the release of latent heat and the invigoration of the updraft. Hence, an understanding of the ice nucleating properties of volcanic ash is important for understanding issues such as the dispersion of volcanic gases which can increase human mortality if advected to populated areas [7].

During the formation of mixed phase clouds significant ice formation normally occurs only after liquid water droplets have formed [2,8]. Freezing of supercooled water droplets can be triggered by INPs via several pathways [9]. Contact freezing involves the collision of an INP with a supercooled droplet upon which the droplet freezes. Condensation freezing describes a sequence of events whereby an aerosol particle takes up water prior to freezing of the condensate. Immersion freezing is caused by an INP suspended in a supercooled water droplet. There is some ambiguity between immersion and condensation freezing, since an aerosol particle could conceivably first serve as a cloud condensation nucleus (CCN) and activate to a cloud droplet. After acting as a CCN, an insoluble component immersed in the cloud droplet may trigger heterogeneous freezing. We take the pragmatic view that when an INP is immersed inside a droplet, it is freezing in the immersion mode and it is in this mode that we study the ice nucleating ability of volcanic ash.

The Soufriere Hills volcano is located on the island of Montserrat in the Caribbean, which is dominated by low level (1–5 km) easterly winds and intermediate level westerly winds (8–18 km), where mean background aerosol concentrations are relatively low due to a lack of local sources [10]. This leads to volcanic eruptions drastically increasing aerosol concentrations over short timescales [11].

The Soufriere Hills volcano had been dormant since the 19^th^ century until 1995, when following extensive seismic activity, it became active again [12]. Between 1995 and 1999, the first phase of eruptive activity was characterised by repeated lava dome growth and collapse, with frequently occurring pyroclastic flows and ash plumes [13]. For the first half of 1999, ash venting was a daily occurrence. A second phase of lava extrusion from 1999–2003 lead to the explosive eruption of July, 2003, when significant sections of the volcanoes dome collapsed. From 2003–2009, several phases (3^rd^ and 4^th^ phase) of explosions, lava extrusion and ash venting occurred at the Soufriere Hills volcano, including a whole dome collapse on the 20^th^ May 2006 [14]. More recently, phase 5 of the volcanoes activity lead to the partial collapse of the dome on the 11^th^ February 2010 (from which the sample used in this study was taken) [15]. Although eruptive activity has slowed since 2010, the Soufriere Hills volcano is currently an important source of episodic aerosol production which may influence cloud formation in the region.

A number of previous studies have established that volcanic ash has the capacity to nucleate ice. Early research suggested a range of volcanic ash samples could act as INPs but these studies made use of fog chambers where the nucleation mode was not constrained and the freezing characteristics were reported with the semi-quantitative measure of threshold freezing temperature [16]. More recent research by Durant et al. [6] has evaluated volcanic ash samples from Chile and Hawaii in the immersion freezing mode giving overall median freezing temperatures of 253.1 K. They also investigated the effects of composition and surface area on the ice nucleating efficiencies of volcanic ash, but found only a weak correlation with freezing temperature. Kulkarni et al. [17] has suggested that composition is of secondary importance compared to crystallographic structure regarding the ice nucleating efficiency of volcanic ash. Fornea et al. [18] determined an average freezing temperature of 254.9 ± 2.0 K for large ash particles (250–300 μm) immersed in droplets. In their study it was also shown that particles in contact with the surface of the droplet froze approximately 7 K warmer than when the particle was fully immersed within a droplet, a similar finding to that of Durant and Shaw [19]. Gibbs et al. [20] recently reported that a rhyolite glass from the Minoan eruption of Santorini in Greece nucleated ice when immersed in water. Ice nucleation by fine ash from the Icelandic Eyjafjallajökull eruption in 2010 has been studied and may have had a significant impact on supercooled cloud glaciation over Europe [4,21,22].

Murray et al. [2] reviewed and reassessed the available laboratory literature data (prior to 2012) for volcanic ash and was only able to quantify the efficiency of ice nucleation for ash from two locations, Eyjafjallajökull and Mount St. Helens in three studies [18,21,22]. The three studies formed a consistent story over the temperature range, but in a more recent study it was shown that ash from other locations had very different ice nucleating abilities [23]. In this study we present an evaluation of the ice nucleating efficiency of an ash sample from the Soufriere Hills volcano on Montserrat in the Caribbean.

## Experimental

The Soufriere Hills ash sample was collected from the surface north east of the volcano’s dome (E297° 50.7’ N16° 45.3’) after the partial collapse of the volcano’s lava dome produced large ash clouds on the 11^th^ February. The ash sample was collected from a metal surface above the ground, removing the risk of contamination from the soil surface. The ash sample was ground in order to make the particulates small enough to suspend in water. The mass fraction of the various crystalline components and amorphous fraction was determined using powder X-ray diffraction (XRD) and Rietveld refinement (see Table 1) [24,25].The specific surface area of the ground ash was determined to be 4.07 ± 0.02 m^2^ g^-1^ using the Brunauer-Emmett-Teller (BET) nitrogen gas absorption method. From this, the surface area of ash per droplet has been calculated using the weight fraction and median droplet volume, in conjunction with the specific surface area.

**Table 1:** Mineralogy of the ash sample (%) used here and the samples used by Schill et al. [23].

Mineral / Sample	Anorthite (Ca-Feldspar)	Enstatite	Cristobalite	Albite (Na-Feldspar)	Tremolite	Quartz	Riebeckite
Soufriere Hills (this study)	45	18.8	16.6	11	5.9	2.7	-
Soufriere Hills (Schill et al., 2015)	10	11	-	71	-	1	7
Fuego (Schill et al., 2015)	36	-	-	64	-	-	-
Oruanui (Schill et al., 2015)	26	-	-	47	-	27	-

Two separate instruments were used to quantify ice nucleation by the Soufriere hills volcanic ash sample over a wide range of temperatures. Both instruments are based on a cold stage upon which droplets are placed, cooled and frozen. They are collectively referred to as the Nucleation by Immersed Particle Instrument (NIPI) suite. Each instrument is designed to operate with a specific range of droplet sizes, where larger droplets contain a greater surface area of INPs and are therefore more likely to freeze at warmer temperatures. For the experiments reported here we used droplets ranging from 0.1 to 1 nanolitres in the nL-NIPI and droplets of one microlitre in μL-NIPI.

We first discuss the nL-NIPI which has been used in several previous studies [26–28]. Briefly, nL-NIPI consists of an aluminium cold stage cooled by liquid nitrogen and temperature is controlled using two embedded cartridge heaters in conjunction with a temperature control unit (Eurotherm 2416); the estimated temperature measurement uncertainty is ±0.2 K. It can be cooled at a defined rate (10 K min^-1^ was used here). Nanolitre volume (30–70 μm diameter) droplets were placed on a hydrophobically coated, siliconised glass cover slip which in turn sits on top of a thermally conductive diamond window through which the droplets are illuminated from below, allowing their phase to be determined with an optical microscope (Olympus BX51). Droplets are generated by first suspending a known mass of ash in a volume of ultra-pure water (18.2 MΩ cm) and then nebulising this suspension into a chamber where they are allowed to settle onto the glass cover slip. The humidity in the chamber is maintained at water saturation in order to prevent condensation or evaporation of water from the droplets. Silicone oil is then placed over the top of the droplets to prevent them from evaporating during the experiment. The oil has previously been shown not to influence heterogeneous freezing by comparison of experiments with and without the oil [24,29].

The μL-NIPI was used to examine the ice nucleation efficiency of the volcanic ash at higher temperatures. This system is described in detail elsewhere [30] and has been used in other studies [26–28,31,32]. A Picus Biohit electronic micropipette was used to pipette 1 μL droplets of ash suspension onto a hydrophobic siliconised glass slide placed onto the temperature controlled plate of a Grant-Asymptote EF600 Stirling engine. The droplets were then cooled at rates of 0.2–2 K min^-1^ with an estimated temperature uncertainty of ±0.4 K. A gentle flow of dry nitrogen was passed over the droplets to prevent ice growth from frozen droplets inducing freezing in neighbouring unfrozen droplets. Whale et al. [30] showed that a dry nitrogen flow using this apparatus had no effect on the freezing measurements; any effect was therefore smaller than the temperature error of the instrument. The droplets were monitored with a digital camera and the freezing temperature of each droplet determined by subsequent analysis of the video.

## Analysis

In this paper we use the time-independent singular description of ice nucleation to describe the efficiency with which ash nucleates ice [2,3,33]. The relationship of the fraction of droplets frozen (*f*_ice_(*T*)) with the density of active sites (*n*_s_(*T*)) is described by:

$$f_{ice}(T)=\frac{n_{ice}(T)}{n}=1-e^{\left(-n_{s}(T)\sigma \right)}$$

Where *n*_ice_(*T*) is the number of frozen droplets at the specified temperature *T*, *n* is the total number of droplets (liquid and frozen), and *σ* is the surface area of ash per droplet. In the microliter experiments, where all droplets were of the same volume (1 μl), *σ* was determined from the mass of ash per droplet and the specific surface area. For the nanolitre experiments, there was a broad droplet size distribution so the method described in Umo et al. [27] was employed.

The parameter, *n*_s_, provides a pragmatic, surface area normalised, measure of the efficiency with which a particular material can nucleate ice. This allows for comparison between different experimental systems and comparison with different ice nucleating materials. It also allows the estimation of the atmospheric ice nucleating particle concentration if the atmospheric abundance of the tested material is known [2]. However, it should be borne in mind that this convenient description of nucleation neglects time dependence. Recent work shows that the time dependence of nucleation is more important for some ice nucleating species compared to others [32,34]. Hence, we also explore the time dependence of this volcanic ash by conducting experiments at various cooling rates.

## Results and Discussion

**Fig 1:**
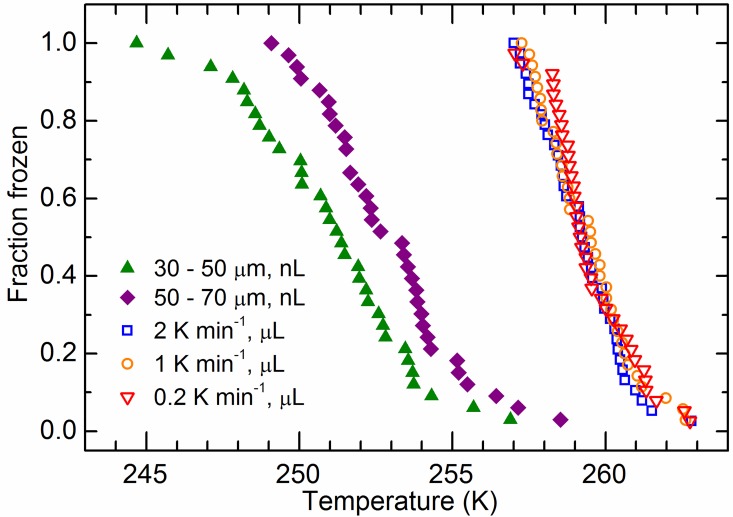
Fraction of droplets frozen as a function of temperature for nanolitre and microlitre volume supercooled water droplets containing Soufriere Hills volcanic ash particles. Droplets with diameters in the range of 30–70 μm (nanolitre volumes) were cooled at 10 K min^-1^ and had ash concentrations of 0.1 wt%. The 1 microlitre droplets had an ash concentration of 0.01 wt% and were cooled at rates from 0.2–2 K min^-1^.

Fig 1 shows the fraction of droplets frozen as a function of temperature for 10 K min^-1^ cooling rate experiments where nanolitre volume droplets contained 0.1 wt% volcanic ash. Freezing occurred heterogeneously from 244.5 to 258.5 K, well above homogeneous freezing (~236 K) [35]. Pure water droplets of similar or larger sizes have been shown to freeze homogeneously using nL-NIPI in the past [26,27]. For the droplets containing ash, higher freezing temperatures are observed for the larger droplet diameters (50–70 μm) compared to the smaller droplets (30–49 μm) as expected, due to a comparatively higher ash surface area present within each droplet. Freezing temperatures for similar experiments performed with microlitre volume droplets with cooling rates varied between 0.2 and 2 K min^-1^ are also shown for comparison in Fig 1. Since the amount of ash in these droplets was 10^3^ to 10^4^ times greater than in the nanolitre droplets they froze at warmer temperatures.

**Fig 2:**
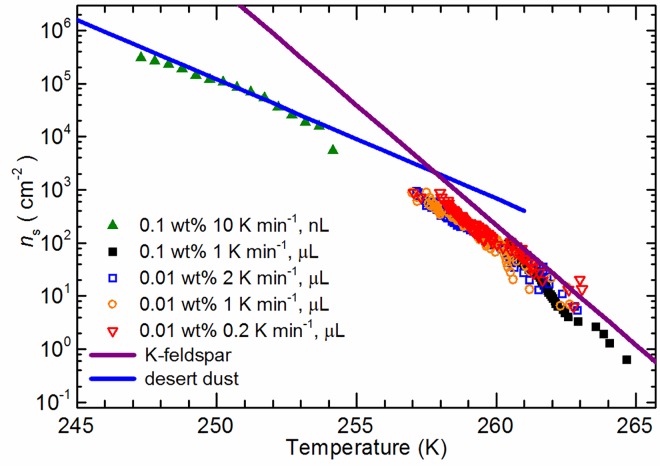
Values of *n*_s_ in units of cm^-2^ as a function of temperature, at concentrations of 0.01-0.1 wt%, for droplets of nanolitre and microlitre volume containing Soufriere Hills volcanic ash. Parameterisations of K-feldspar taken from Atkinson et al. [31] and desert dusts taken from Niemand et al. [36] are included for comparison.

Values of *n*_s_ are shown in Fig 2 for the nanolitre and microlitre experiments. The values of *n*_s_ from the two sets of experiments are consistent with one another. In addition, *n*_s_ from microlitre experiments with both 0.01 and 0.1 wt% ash were also in good agreement probing a total mass range of 1x10^-6^ – 1x10^-10^ g (including the nanolitre experiments), indicating that *n*_s_ is not dependent on the amount of material in the droplet or the size of a droplet. This is important because in order to access small, but atmospherically relevant, values of *n*_s_ we have to have many thousands of particles per droplet. This contrasts with the situation in the atmosphere where each particle (or aggregate of particles) is capable of acting as a CCN and becoming immersed in an individual droplet. The lack of dependence of *n*_s_ on the amount of material in a droplet or the size of the droplet indicates that processes such as particle aggregation do not significantly reduce the surface area available to nucleation in our experiments.

There is also no observable dependence of *n*_s_ on cooling rate, which was varied between 0.2 and 2 K min^-1^ in the microlitre experiments and 10 K min^-1^ in the nanolitre experiments. Herbert et al. [32] show that the shift in temperature between fraction frozen curves on changing cooling rate is determined by the slope of the nucleation rate of a single component with temperature (−dln(*J*_s,i_)/d*T* = *λ*). Smaller values of *λ* correspond to stronger time dependence. Given our temperature uncertainty and the statistical spread in the fraction frozen curves in Fig 1 we estimate that *λ* was larger than 4 K^-1^ in the observed temperature range. For a single component material the magnitude of *λ* is equal to the slope of dln(*n*_s_,i)/d*T*, but for a multiple component material dln(*n*_s_,i)/d*T* is smaller than *λ*. The value of dln(*n*_s_,i)/d*T* from Fig 2 for the steepest part of the curve is 1 K^-1^, which is smaller than *λ*, indicating that this material is best described as a multiple component species. This implies that there is strong particle-to-particle variability in the ice nucleating ability of this volcanic ash. This contrasts with a single component material (such as kaolinite KGa1b) where each particle has a similar ability to nucleate ice [32].

The weak time dependence (large *λ*) is consistent with the fact the microlitre and nanolitre *n*_s_ values overlap despite being determined from experiments with different cooling rates. In contrast, Herbert et al. [32] estimated that Eyjafjallajökull ash and Mount St Helens ash had a *λ* value of about 0.6 K^-1^, which would cause a shift in the mean freezing temperature of > 3 K for a factor of 10 change in cooling rate. This is strikingly different to the minimal time dependent behaviour observed in the present study which indicates that the ice nucleating component of the Soufriere Hills ash is distinct to the Eyjafjallajökull ash and Mount St. Helens ash samples. The Soufriere Hills ash sample has a *λ* more like that of biological ice nucleators such as *Pseudomonas syringae* or inorganic nucleators such as Arizona Test Dust and K-feldspar [32]. The X-ray diffraction analysis (Table 1) indicates that there is a significant component of Ca and Na feldspars; hence it is possible that it is the feldspar component in Soufriere Hills ash which nucleates ice.

The *n*_s_ values for Soufriere Hills ash are also compared with literature data for microcline (K-feldspar) and desert dust in Fig 2. The ash sample nucleates ice with *n*_s_ values very close to that of microcline [31] between 257 and 265 K and is similar to the Niemand et al. [36] desert dust parameterisation below 256 K. More recent measurements of ice nucleation by a range of feldspars suggest *n*_s_ curves off and reaches a limiting value below about 250 K [37,38], perhaps more consistent with the trend displayed by Soufriere Hills volcanic ash in this study. Overall, this particular volcanic ash has a nucleating ability comparable to mineral dusts from deserts.

**Fig 3:**
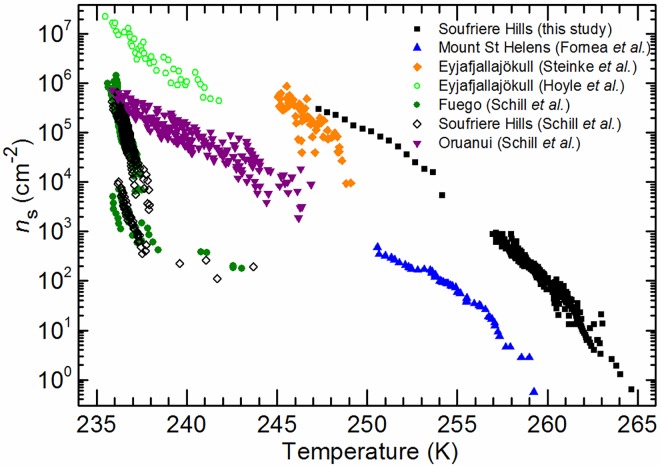
Values of *n*_s_ in units of cm^-2^ as a function of temperature, for ice nucleation by volcanic ashes in the immersion/condensation modes from this study and the literature [18,21–23].

We now compare the results for our ash sample to *n*_s_ values for volcanic ash from the literature in Fig 3. In general, the available data indicates that the ice nucleating ability of volcanic ash samples is highly variable. Our sample of the Soufriere Hills volcanic ash is one of the most ice-active ashes used in any of the available immersion/condensation mode studies. In contrast to our data, another sample from the Soufriere Hills studied by Schill et al. [23] was very poor at nucleating ice, with nucleation occurring close to the homogeneous limit. There were some mineralogical differences between the Soufriere Hills ashes used in our study and that of Schill et al. [23]. Our sample contained a greater proportion of Anorthite and less Albite compared to their sample. Schill et al. [23] also report that their sample contained 11% amorphous material, whereas our sample contained no detectable amorphous material. Our ash sample was collected after a dome collapse eruption on the 11^th^ of February 2010, while Schill et al. [23] report that their sample resulted from an explosive eruption in January 2010 [15]. These compositional differences may be due to the different eruption types, with dome collapse eruptions leading to less amorphous material compared to the freshly erupted magma from an explosive eruption, which is rapidly quenched. The quartz polymorph cristobalite (present in our sample) is a common product of lava dome eruptions but would not be found as a product from explosive eruptions [39], such as the ash studied in Schill et al. [23]. The ice nucleating activity of ash may therefore differ depending on the type of eruption and inherently also on composition. A direct comparison study of the ice nucleating ability of ash from different lava dome and explosive eruption types could evaluate this possibility. We also note that Schill et al. [23] agitated their suspensions for 12 hours prior to their experiments whereas we conducted the experiments within a matter of minutes of suspending the dust. In a separate study we have found that an albite sample can nucleate ice very efficiently when first immersed in water, but this activity decreases dramatically with time [40]. It is possible something similar is occurring here where an ice active component of the ash is deactivated with time spent in water. Schill et al. [23] report *n*_s_ values for two other ash samples, one of which was similarly inactive to their Soufriere Hills sample (Fuego) and another from New Zealand (Oruanui) which was much more active. It should be noted that the crystalline fraction of both of these samples were primarily composed of feldspars, but the ashes were also comprised of a significant amorphous component. This amorphous fraction may account for the lower ice nucleating activity as previously hypothesised by Kulkarni et al. [17]. However, the New Zealand ash sample was mineralogically distinct, also containing a significant amount of crystalline quartz as well as being made of 59% amorphous material. Schill et al. [23] suggest that quartz might be responsible for its ice nucleating ability.

Feldspars are known to be sensitive to exposure to acid with their surfaces rapidly transforming into amorphous silicates and clay minerals [31]. Kulkarni et al. [17] also suggest that the ‘crystallinity’ of ashes might be affected by acid coatings which might influence ice nucleating activity. It has been shown that samples in which there is a significant feldspar fraction reduce in ice nucleation activity on exposure to acid, which is presumably due to the conversion of the highly ice active feldspar surfaces to less active materials [41]. Since volcanic plumes are characterised by high concentrations of the acid gas SO_2_ in the presence of water vapour, it is possible that aerosolised volcanic ash may be deactivated when acid collects on their surfaces. In this study we ground the ash sample to break up the largest of the particles. This may also have exposed fresh highly active feldspar surfaces, hence the results presented here may represent an upper limit to the ice nucleating ability of Soufriere Hills ash, although a similar grinding process was also used by Schill et al. [23] who reported very low activity for two out of three of their volcanic ash samples. More work is needed to quantify the acid passivation of volcanic ash under relevant conditions as well as the role that mineralogy and amorphous content plays in its ice nucleating ability.

## Summary and Conclusions

We present an experimental evaluation of the ice nucleating efficiency of ash from the Soufriere Hills volcano. In this study ash particles were immersed in droplets and the efficiency with which they nucleated ice was quantified. Comparison with literature data for other ashes shows that there are substantial differences in ashes from different sources and even from different eruptions of the same volcano. This contrasts with the view expressed previously that many ashes have a similar capacity to nucleate ice [2] or that the majority of ashes have an onset freezing threshold within 10 K [6]. We suggest that the ice nucleating ability of Soufriere Hills ash may be due to the presence of Na Feldspars. Given that feldspars may be important for ice nucleating in Soufriere Hills volcanic ash, there is the potential that the ash’s ice nucleating ability may be strongly reduced by the presence of sulphuric acid, as feldspars are chemically converted to other materials when in contact with acid. The samples used here were ground in order to make the particles small enough to suspend in water. This may have exposed fresh surfaces upon which ice nucleation could occur. More work is needed in order to address this issue. Nevertheless, this study together with previous work demonstrates that volcanic ashes have diverse ice nucleating properties and that Soufriere Hills ash has the potential to be a source of effective atmospheric INPs.

## References

[1] Lohmann U, Feichter J (2005) Global indirect aerosol effects: a review. Atmospheric Chemistry and Physics 5: 715–737.

[2] Murray BJ, O'Sullivan D, Atkinson JD, Webb ME (2012) Ice nucleation by particles immersed in supercooled cloud droplets. Chemical Society reviews 41: 6519–6554. 10.1039/c2cs35200a 22932664

[3] Hoose C, Mohler O (2012) Heterogeneous ice nucleation on atmospheric aerosols: a review of results from laboratory experiments. Atmospheric Chemistry and Physics 12: 9817–9854.

[4] Bingemer H, Klein H, Ebert M, Haunold W, Bundke U, Herrmann T, et al (2012) Atmospheric ice nuclei in the Eyjafjallajökull volcanic ash plume. Atmospheric Chemistry and Physics 12: 857–867.

[5] Hobbs PV, Fullerton CM, Bluhm GC (1971) Ice Nucleus Storms in Hawaii. Nature-Physical Science 230: 90–91.

[6] Durant AJ, Shaw RA, Rose WI, Mi Y, Ernst GGJ (2008) Ice nucleation and overseeding of ice in volcanic clouds. Journal of Geophysical Research-Atmospheres 113: 13.

[7] Schmidt A, Ostro B, Carslaw KS, Wilson M, Thordarson T, Mann GW, et al (2011) Excess mortality in Europe following a future Laki-style Icelandic eruption. Proceedings of the National Academy of Sciences of the United States of America 108: 15710–15715. 10.1073/pnas.1108569108 21930954 PMC3179041

[8] de Boer G, Morrison H, Shupe MD, Hildner R (2011) Evidence of liquid dependent ice nucleation in high-latitude stratiform clouds from surface remote sensors. Geophys Res Lett 38.

[9] Vali G (1985) Nucleation Terminology. Bulletin of the American Meteorological Society 66: 1426–1427.

[10] Bonadonna C, Loughlin SC, Norton GE, Young SR (2002) Tephra fallout in the eruption of Soufriere Hills Volcano, Montserrat. The eruption of Soufriere Hills Volcano, Montserrat from 1995 to 1999: Kokelaar.

[11] Mather TA, Pyle DM, Oppenheimer C (2003) Tropospheric Volcanic Aerosol. Geophysical monograph 139: 189–212.

[12] Francis P, Druitt TH, Kokelaar BP (2002) The eruption of Soufrière Hills volcano, Montserrat, from 1995 to 1999. London: Geological Society.

[13] Druitt TH, Young SR, Baptie B, Bonadonna C, Calder ES, Clarke AB, et al (2002) Episodes of cyclic Vulcanian explosive activity with fountain collapse at Soufrière Hills Volcano, Montserrat. Geological Society, London, Memoirs 21: 281–306.

[14] Cole PD, Smith P, Komorowski J-C, Alfano F, Bonadonna C, Stinton A, et al (2014) Chapter 4 Ash venting occurring both prior to and during lava extrusion at Soufrière Hills Volcano, Montserrat, from 2005 to 2010. Geological Society, London, Memoirs 39: 71–92.

[15] Cole P, Bass V, Christopher T, Eligon C, Fergus M, Gunn L, et al. (2010) Report to the Scientific Advisory Committee on Montserrat Volcanic Activity—Report on Activity between 15 August 2009 and 28 February 2010 Part 1—Main Report. Montserrat Volcano Observatory Open File Report 10-01a

[16] Mason BJ (1971) The physics of clouds. Oxford: Clarendon Press.

[17] Kulkarni G, Nandasiri M, Zelenyuk A, Beranek J, Madaan N, Devaraj A, et al (2015) Effects of crystallographic properties on the ice nucleation properties of volcanic ash particles. Geophysical Research Letters 42: 3048–3055.

[18] Fornea AP, Brooks SD, Dooley JB, Saha A (2009) Heterogeneous freezing of ice on atmospheric aerosols containing ash, soot, and soil. Journal of Geophysical Research-Atmospheres 114.

[19] Durant AJ, Shaw RA (2005) Evaporation freezing by contact nucleation inside-out. Geophysical Research Letters 32: 4.

[20] Gibbs A, Charman M, Schwarzacher W, Rust AC (2015) Immersion freezing of supercooled water drops containing glassy volcanic ash particles. GeoResJ 7: 66–69.

[21] Steinke I, Mohler O, Kiselev A, Niemand M, Saathoff H, Schnaiter M, et al (2011) Ice nucleation properties of fine ash particles from the Eyjafjallajökull eruption in April 2010. Atmos Chem Phys 11: 12945–12958.

[22] Hoyle CR, Pinti V, Welti A, Zobrist B, Marcolli C, Luo B, et al (2011) Ice nucleation properties of volcanic ash from Eyjafjallajökull. Atmospheric Chemistry and Physics 11: 9911–9926.

[23] Schill GP, Genareau K, Tolbert MA (2015) Deposition and immersion-mode nucleation of ice by three distinct samples of volcanic ash. Atmos Chem Phys 15: 7523–7536.

[24] Broadley SL, Murray BJ, Herbert RJ, Atkinson JD, Dobbie S, Malkin TL, et al (2012) Immersion mode heterogeneous ice nucleation by an illite rich powder representative of atmospheric mineral dust. Atmospheric Chemistry and Physics 12: 287–307.

[25] Hillier S (2003) Quantitative analysis of clay and other minerals in sandstones by X-ray powder diffraction (XRPD). Special Publication- International Association of Sedimentologists 34: 213–252.

[26] O'Sullivan D, Murray BJ, Malkin TL, Whale TF, Umo NS, Atkinson JD, et al (2014) Ice nucleation by fertile soil dusts: relative importance of mineral and biogenic components. Atmospheric Chemistry and Physics 14: 1853–1867.

[27] Umo NS, Murray BJ, Baeza-Romero MT, Jones JM, Lea-Langton AR, Malkin TL, et al (2015) Ice nucleation by combustion ash particles at conditions relevant to mixed-phase clouds. Atmos Chem Phys 15: 5195–5210.

[28] O'Sullivan D, Murray BJ, Ross JF, Whale TF, Price HC, Atkinson JD, et al (2015) The relevance of nanoscale biological fragments for ice nucleation in clouds. Scientific Reports 5. 10.1038/srep08082 PMC4308702 25626414

[29] Murray BJ, Broadley SL, Wilson TW, Atkinson JD, Wills RH (2011) Heterogeneous freezing of water droplets containing kaolinite particles. Atmospheric Chemistry and Physics 11: 4191–4207.

[30] Whale TF, Murray BJ, O'Sullivan D, Wilson TW, Umo NS, Baustian KJ, et al (2015) A technique for quantifying heterogeneous ice nucleation in microlitre supercooled water droplets. Atmospheric Measurement Techniques 8: 2437–2447.

[31] Atkinson JD, Murray BJ, Woodhouse MT, Whale TF, Baustian KJ, Carslaw KS, et al (2013) The importance of feldspar for ice nucleation by mineral dust in mixed-phase clouds. Nature 498: 355–358. 10.1038/nature12278 23760484

[32] Herbert RJ, Murray BJ, Whale TF, Dobbie SJ, Atkinson JD (2014) Representing time-dependent freezing behaviour in immersion mode ice nucleation. Atmospheric Chemistry and Physics 14: 8501–8520.

[33] Connolly PJ, Mohler O, Field PR, Saathoff H, Burgess R, Choularton T, et al (2009) Studies of heterogeneous freezing by three different desert dust samples. Atmos Chem Phys 9: 2805–2824.

[34] Wright TP, Petters MD (2013) The role of time in heterogeneous freezing nucleation. Journal of Geophysical Research-Atmospheres 118: 3731–3743.

[35] Murray BJ, Broadley SL, Wilson TW, Bull SJ, Wills RH, Christenson HK, et al (2010) Kinetics of the homogeneous freezing of water. Physical Chemistry Chemical Physics 12: 10380–10387. 10.1039/c003297b 20577704

[36] Niemand M, Möhler O, Vogel B, Vogel H, Hoose C, Connolly P, et al (2012) A Particle-Surface-Area-Based Parameterization of Immersion Freezing on Desert Dust Particles. Journal of the Atmospheric Sciences 69: 3077–3092.

[37] Peckhaus A, Kiselev A, Hiron T, Ebert M, Leisner T (2016) A comparative study of K-rich and Na/Ca-rich feldspar ice-nucleating particles in a nanoliter droplet freezing assay. Atmos Chem Phys 16: 11477–11496.

[38] Niedermeier D, Augustin-Bauditz S, Hartmann S, Wex H, Ignatius K, Stratmann F (2015) Can we define an asymptotic value for the ice active surface site density for heterogeneous ice nucleation? Journal of Geophysical Research: Atmospheres 120: 5036–5046.

[39] Baxter PJ, Bonadonna C, Dupree R, Hards VL, Kohn SC, Murphy MD, et al (1999) Cristobalite in Volcanic Ash of the Soufriere Hills Volcano, Montserrat, British West Indies. Science 283: 1142–1145. 10024235 10.1126/science.283.5405.1142

[40] Harrison AD, Whale TF, Carpenter MA, Holden MA, Neve L, O'Sullivan D, et al (2016) Not all feldspar is equal: a survey of ice nucleating properties across the feldspar group of minerals. Atmos Chem Phys Discuss 2016: 1–26.

[41] Wex H, DeMott PJ, Tobo Y, Hartmann S, Rösch M, Clauss T, et al (2014) Kaolinite particles as ice nuclei: learning from the use of different kaolinite samples and different coatings. Atmos Chem Phys 14: 5529–5546.

